# Gut microbiome associated with APC gene mutation in patients with intestinal adenomatous polyps

**DOI:** 10.7150/ijbs.37399

**Published:** 2020-01-01

**Authors:** Siyuan Liang, Yan Mao, Ming Liao, Yansong Xu, Yingchun Chen, Xiaoliang Huang, Chuangyi Wei, Changtao Wu, Qiuyan Wang, Xiaoyan Pan, Weizhong Tang

**Affiliations:** 1 Guangxi Medical University Affiliated Tumor Hospital, Nanning, Guangxi, 530021, China.; 2 Guangxi Medical University, Nanning, Guangxi, 530021, China.; 3 The First Affiliated Hospital of Guangxi Medical University, Nanning, Guangxi, 530021, China.; 4 Oncology Department, Nanning Second People's Hospital, The Third Affiliated Hospital of Guangxi Medical University, Nanning, Guangxi, 530031, China.; 5 Guangxi Key Laboratory of Genomic and Personalized Medicine, Nanning, Guangxi, 530021, China.; 6 Department of Reproductive Center, The First Affiliated Hospital of Guangxi Medical University, Nanning, Guangxi, 530021, China.

**Keywords:** Colorectal cancer, Gut microbiome, Metagenomic analyses, Metabolism analyses, APC mutation.

## Abstract

**Background:** The 'adenoma-carcinoma sequence' is a well-recognized model of colorectal cancer (CRC) development. However, the interaction between gut microbiota and genetic variation in the initiation of CRC is not clear. Our study attempts to demonstrate the relationship between gut microbiota and host genetics in patients with intestinal adenomatous polyps.

**Method:** The entire exon region of the APC gene was sequenced in 35 patients with pathologically diagnosed adenomatous polyps. Patients with highly pathogenic APC mutation were classified as the case group, while the others were classified as the control group. The patients'stool and serum samples were respectively collected for metagenomics and metabolomics measurements.

**Results:** In the analysis of gut microbiome, there were three most important species, in which Fusobacterium_mortiferum was significantly increased while Faecalibacterium_prausnitzii and Bifidobacterium_pseudocatenulatum were significantly decreased in the case group. The significantly low abundance of the Photosynthesis pathway in patients with APC mutation was due to the low abundance of species Faecalibacterium_prausnitzii and Bifidobacterium_pseudocatenulatum. Moreover, there were two clusters of KEGG pathways correlated with two clusters of species characterized by Faecalibacterium_prausnitzii and Fusobacterium_mortiferum. As to serum metabolomics, the abundance of (R)-3-Hydroxybutyric acid and 2-Hydroxyphenethylamine were significantly higher in patients with APC mutation, while the abundance of 1-Aminocyclopropanecarboxylic acid,7-Ketocholesterol, DL-lactate, and L-Pyroglutamic acid were significantly higher in controlgroup. After analyzing the metabolome and microbiome data by sparCCmethod, we found that there was a significantly negative correlation between the abundance of Faecalibacterium_prausnitzii and Fusobacterium_mortiferum, and a significantly positive correlation between Faecalibacterium_prausnitzii abundance and the steroid hormone Hydrocortisone (Cortisol) in serum.

**Conclusions:** Host's APC mutation was closely related to the changes of gut microbiota and serum metabolites, and some species of gut microbiome like Faecalibacterium_prausnitzii and Fusobacterium_mortiferum might have the potential to predict the development of CRC from intestinal adenomatous polyps.

## Introduction

Colorectal cancer (CRC) is the third most common cancer and ranks fourth as a cause of cancer-related death worldwide 1. It is estimated that 140,250 new cases and 50,630 deaths will occur in 2018 in the United States 2. The well-recognized “adenoma-carcinoma sequence” is known to play a significant role in CRC development 3. The adenomas are the most common premalignant precursor lesions of almost all the sporadic CRCs. It is estimated that adenomatous polyps develop in up to 40% of people over the age of 60 4. The transformation rate of adenomatous polyps into cancer is about 0.25% per year 5. The underlying molecular mechanisms driving the transformation include the accumulating somatic and germ-line mutations. Inactivating mutations of the adenomatous polyposis coli (APC) gene is regarded as a trigger of the “adenoma-carcinoma sequence”. Loss of APC gene function facilitates the accumulation of β-catenin, resulting in aberrant cellular proliferation, leading to the formation of adenomatous polyposis 6.

APC is located at 5q21-q22, which contains 15 exons and codes a 310-kDa protein. Mutation in the APC is the most common genetic variation in CRC. There were more than 80% of CRC patients with APC mutation 7. Up to now, at least 3000 different pathogenic APC mutations have been identified. The majority of APC mutations often fall within the mutation cluster region (MCR, codons 1,286-1,513) of the APC, which often results in a truncated APC protein 8. The role of truncated APC protein in tumorigenesis of CRC is complicated. In recent years, it has been increasingly recognized that genetic factors and environmental and their interaction are implicated in the tumorigenesis of CRC 9.

The human gastrointestinal tract harbors a complex population of microorganisms, the gut microbiota, which together with make up a population exceed 1014 10. Many diseases were demonstrated to have links with the change of gut microbiota, such as metabolic disorders, gastrointestinal disorders, neuropsychological diseases and so on 11. With the constant studying of the relationship among gut microbiome, host and human diseases, accumulated evidence indicates that there is a certain relationship between host genetics and gut microbiota, and the change of some certain bacteria may be influenced by the mutation of host genes 12. This kind of changes may work together with the mutation of host genes to promote the development of disease 13. Gut microbiota has been reported in interaction with genetic in contributing to the genetic paradigm of the “adenoma-carcinoma sequence” 14. Bacterial drivers are involved in the initiation of precancerous lesions and accumulation of a cascade of gene mutations during the “adenoma-carcinoma sequence”. With the development of the tumor, the passenger bacteria with a competitive advantage in the tumor niche may replace the bacterial drivers gradually. Hence, identifying the gut microbiota associated with a gene mutation in the initiation of CRC may provide new insight into the tumorigenesis and early prevention of CRC.

In this study, we are aiming at exploring the relationship among inactivating APC mutation, the change of gut microbiome and the serum metabolites in patients with intestinal adenomatous polyps, and expecting these associations may partially explain a role of the gut microbiome in the conversion of APC mutant intestinal adenomatous polyps to CRC.

## Materials and Methods

### Patients

The study was authorized by the First Affiliated Hospital of Guangxi Medical University (Nanning, China).In this study, we enrolled 35 patients with adenomatous polyps in the First Affiliated Hospital of Guangxi Medical University. The enrollment criteria were as follows: 1) No antibiotics were taken within two months, 2) Age ranged from 40 to 69 years, 3) Local natives living in Guangxi for more than five years, 4) Those with other intestinal diseases were excluded. Information about the patients'height, weight, age, and other clinical data was collected (Table S1). All the patients were diagnosed pathologically and provided the informed consent.

Fecal samples were taken at least one month after the patient had done a colonoscopy. We took the stool immediately into the ice box and stored it in a -80℃ refrigerator within 2 hours. The serum was collected on the morning of the stool collection. Fasting blood collection was taken in the morning, and the sample was stationary for 30 minutes after taking 5ml whole blood with EDTA tube. The blood was then centrifuged for 10 minutes at 3600 rpm. Finally, the serum was drawn into a 2ml EP tube and immediately stored in a -80℃ refrigerator. And adenomatous polyp tissue consists of two parts: 13 samples came from fresh tissue and others were FFPE cuts.

### DNA Extraction

The patients'fresh frozen and FFEP adenomatous polyp tissues were extracted according to the standard procedures of E.Z.N.A. Tissue DNA Kit and E.Z.N.A.® FFEP DNA Kit respectively.

### APC amplicons sequencing

Amplicon Cancer panel (Shanghai Biowing Applied Biotechnology Ltd.) was prepared with recommended DNA amount (150ng for fresh frozen material, 250ng for FFPE samples). The panel includes 76 amplicons of about 200bp length, targeting all exons in the APC gene (Table S2). The libraries were constructed with three-round PCR. Each amplicon added index sequences for sample multiplexing (i5 and i7) PCR products were purified using AMPure XP beads (Beckman Coulter, Brea, CA), quantified, normalized to 4 nM to ensure equal library representation in the pooled sample and sequenced on an X-10 instrument (Illumina) with PE150 model.

All the sequencing reads were separated according to the corresponding samples based on index sequences by using FASTX-Toolkit with a parameter that mismatch base of index sequence was less than 1. After that, the index and adapter sequence was trimmed out by using cutadapt software, generating target sequences for each sample. Then, sequencing reads were mapped against the reference genome (Grch38) to generate the sam file by BWA (v0.7.12). Indels in sequence alignment files were left-aligned and local realignmentaroundIndels was done with the RealignerTargetCreator and the IndelRealigner tools from the Genome Analysis Toolkit (GATK, version 2.4-9). Base quality score recalibration was performed. Duplicate mapping and marking was not deemed suitable for amplicon sequencing and thus omitted. Unified Genotyper from the GATK (version 2.4-9) was used for variant calling. Variant Effect Predictor (McLaren et al., 2016.DOI: 10.1186/s13059-016-0974-4) was used for variant annotation.

### Metagenomic measurement

DNA preparation: DNA concentrations were measured with the NanoDrop 2000 (Thermo Fisher Scientific), and sheared with Covaris S220 Sonicator (Covaris) to the target of 300-400 average bp size. Fragmented DNA was purified using Sample Purification Beads (Illumina). Adapter-ligated libraries were prepared with the TruSeq Nano DNA Sample Prep Kits (Illumina) according to Illumina-provided protocol.

DNA sequencing: DNA concentrations of the resulting sequencing libraries were measured with the Qubit 2.0 fluorometerdsDNA HS Assay (Thermo Fisher Scientific). Quantities and sizes of the resulting sequencing libraries were analyzed using Agilent BioAnalyzer 2100 (Agilent). The libraries were used in cluster formation on an IlluminacBOT cluster generation system with HiSeq X HD PE Cluster Kits (Illumina). Paired-end sequencing is performed using an IlluminaHiSeq X following Illumina-provided protocols for 2x150 paired-end sequencing.

### Metabolomic measurement

UHPLC (1290 Infinity LC, Agilent Technologies) coupled to a quadrupole time-of-flight (AB SciexTripleTOF 6600) was used to analyze the serum sample of the 35 patients.

A 2.1 mm × 100 mm ACQUIY UPLC BEH 1.7 μm column (waters, Ireland) was used to analyse our 35 samples. In both ESI positive and negative modes, A (25 mM ammonium acetate and 25 mM ammonium hydroxide in water) and B (acetonitrile) constitute the mobile phase. The gradient settings was 85%B(acetonitrile) for 1 min and then linearly turn to 65% in 11 min, after that reduced to 40% in 1min and kept for 4 min, and then back to 85% in 0.1 min, with a re-equilibration period for 5 min employed.

The ESI source conditions are formulated as follows: The ion source Gas1 (Gas1) was set to 60, the ion source Gas2 (Gas2) was set to 60, the source temperature was set to 600 °C, the curtain gas (CUR) was set to 30, and the IonSpray voltage floating (ISVF) was set to ±5500 V. High sensitivity mode selected information-related acquisition (IDA) was used to acquire the product scan. The collision energy (CE) was 35 V with ±15 eV. Declustering potential (DP) was fixed at ±60 V. The raw MS data (wiff.scan files) were changed to MzXML by ProteoWizardMSConvert and LCMS was used for feature detection and proofreading. Then the metabolites were matched with standards database and the raw abundance was obtained for statistical analysis.

### Statistical analysis

A total of 410G bases of metagenomesequence was obtained (SUB4251004, the data will be released at the end of December). The amount of sequence collected for each sample is summarized in Table S3. We next identified and removed human reads from the quality filtered reads. The bacterial abundance was quantified by MetaPhlAn 2.0, and the KEGG or MetaCyc pathway was quantified by HUMAnN2. The raw abundance of bacterial genera, microbial pathways and metabolites was normalized to relative abundance by total sum scaling. We filtered out the bacterial genera with max abundance <1% across all samples or present in <10% of all samples, and filtered out the pathways or metabolites with max abundance <0.1% across all samples or present in <10% of all samples. The raw abundance of all the bacterial genera was reformatted for input into LEfSe via the Huttenhower Lab Galaxy Server (https://huttenhower.sph.harvard.edu/galaxy/root). This algorithm performed nonparametric statistical testing and then differentially ranked the abundant taxa by their linear discriminate analysis (LDA) log-scores. Differentially abundant taxa that were statistically significant using an alpha of 0.05, and LDA log-scores exceeding +/-2.0 were visually represented as bar plots. We used the Jensen-Shannon distance to measure the distance between samples for the bacterial abundance data. The multidimensional scaling (MDS) and the permutational multivariate analysis of variance (PERMANOVA) analyses were performed by R packages “metaMDS”and “adonis”. The MDS plots with the representation of point classes were made by R packages “ade4” and “ggplot2”. The Wilcoxon rank-sum test was used to identify the significant microbiomes, microbial pathways or serum metabolites between the APC mutation group and no-APC mutation group. The Spearman correlation was used to evaluate the relationship between the relative abundance of species and pathways.

The species or metabolites that were significant discriminators were categorized into two groups: those that were more abundant in patients without APC mutation (potentially beneficial), and those that were more abundant in patients with APC mutation (potentially harmful). The relative abundances of these significant species or metabolites were summed for each category, and then the difference between the sums was calculated as the risk index value. Receiving Operating Characteristic (ROC) curves were plotted and the area under the curve (AUC) values were computed by the risk index.Correlation analysis was performed using SparCC on the relative abundance of all the serum metabolites and the significant speices^15^. Pseudo p-values were calculated using 100 randomized sets. Networks of correlations were visualized using Cytoscape v3.1.0 16. For easier interpretation, final network visualization was confined to the nodes and edges with P value <0.05 and Spearman correlation coefficients >0.25.

## Results

### Characteristics of patients with adenomatous polyp

Thirty-five patients with adenomatous polyp have detected 0 to 12 mutations in APC exon sequencing. These mutations were annotated by The Ensemble Variant Effect Predictor software and were divided into four classes: high, moderate, low, modifier (Figure S1). Only the highly pathogenic mutation is assumed to have a high (disruptive) impact in the protein, probably causing protein truncation, loss of function or triggering nonsense-mediated decay. Therefore, patients were divided into two groups based on the presence or absence of highly pathogenic mutations (Figure S2). Patents divided into the case group with highly pathogenic mutations of the APC gene (N=13), and the control group without highly pathogenic mutations of the APC gene (N=22). The baseline characteristics between the two groups were shown in Table 1.

### Microbial Community Patterns in Patients with APC mutation

**Figure 1:**
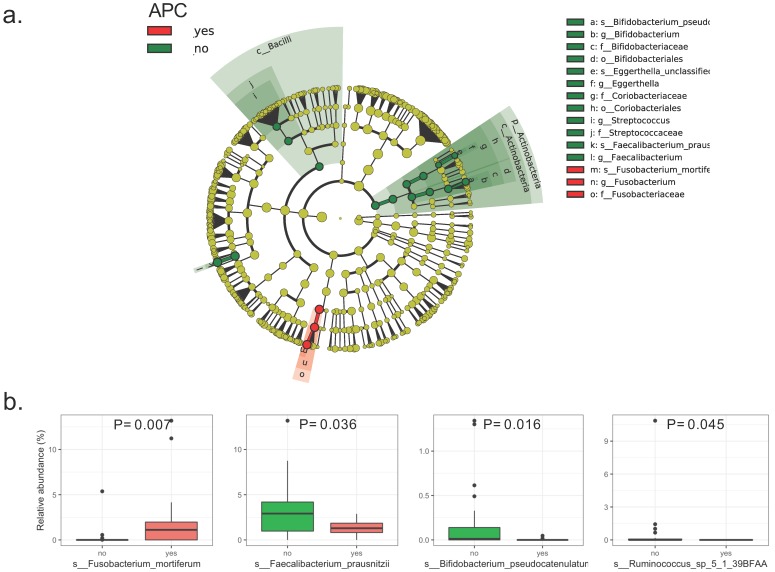
** Bacterial composition in the fecal samples from patients with intestinal adenomatous polyps.** The case group is the APC gene mutation group (red) and the control group is non-APC gene mutation group (green).(a). Cladograme from LEfSe analysis indicated the differentially abundant microbial clades between the two groups. The grey circles represented the non-significant microbial clades; (b). Relative abundance of each significant specie in the two groups. Only the species with P< 0.05 by Wilcoxon rank-sum test were present.

The LEfSe analysis of raw abundances of fecal microbiomes in patients with intestinal adenomatous polyps discovered 4 of 98 genera significantly differed between the case group and the control group. The differentially abundant microbial clades between the two groups covered three families including Ruminococcaceae, Bifidobacteriaceae, Fusobacteriaceae, in which the Ruminococcaceae family included Ruminococcus and Faecalibacteriumgenus (Figure 1a). Four species were showing a significant difference between the two groups. Compared to the patients without APC mutation, the patients with APC mutation had lower relative abundances of Faecalibacterium_prausnitzii, Bifidobacterium_pseudocatenulatum and Ruminococcus_sp_5_1_39BFAA, but the higher relative abundance of Fusobacterium_mortiferum (Figure 1b). The LDA scores from LEfSe analysis were present for each significant taxa in Figure S3. Random forest was used to identify the important species that best-distinguished patients with APC mutation from patients without APC mutationand predicted the status of APC with an accuracy of 78%. The most important species was Fusobacterium_mortiferum followed by Faecalibacterium_prausnitzii and Bifidobacterium_pseudocatenulatum (Figure S4). Based on the MDS of the Jensen-Shannon distance, the patients with APC mutation cannot be separated from patients without APC mutation, by the relative abundance of all the fecal microbiomes in the taxonomic level of species, genus or family, except the order level (Figure S5).

### Analysis of Fecal Microbial Pathways and APC mutation

**Figure 2:**
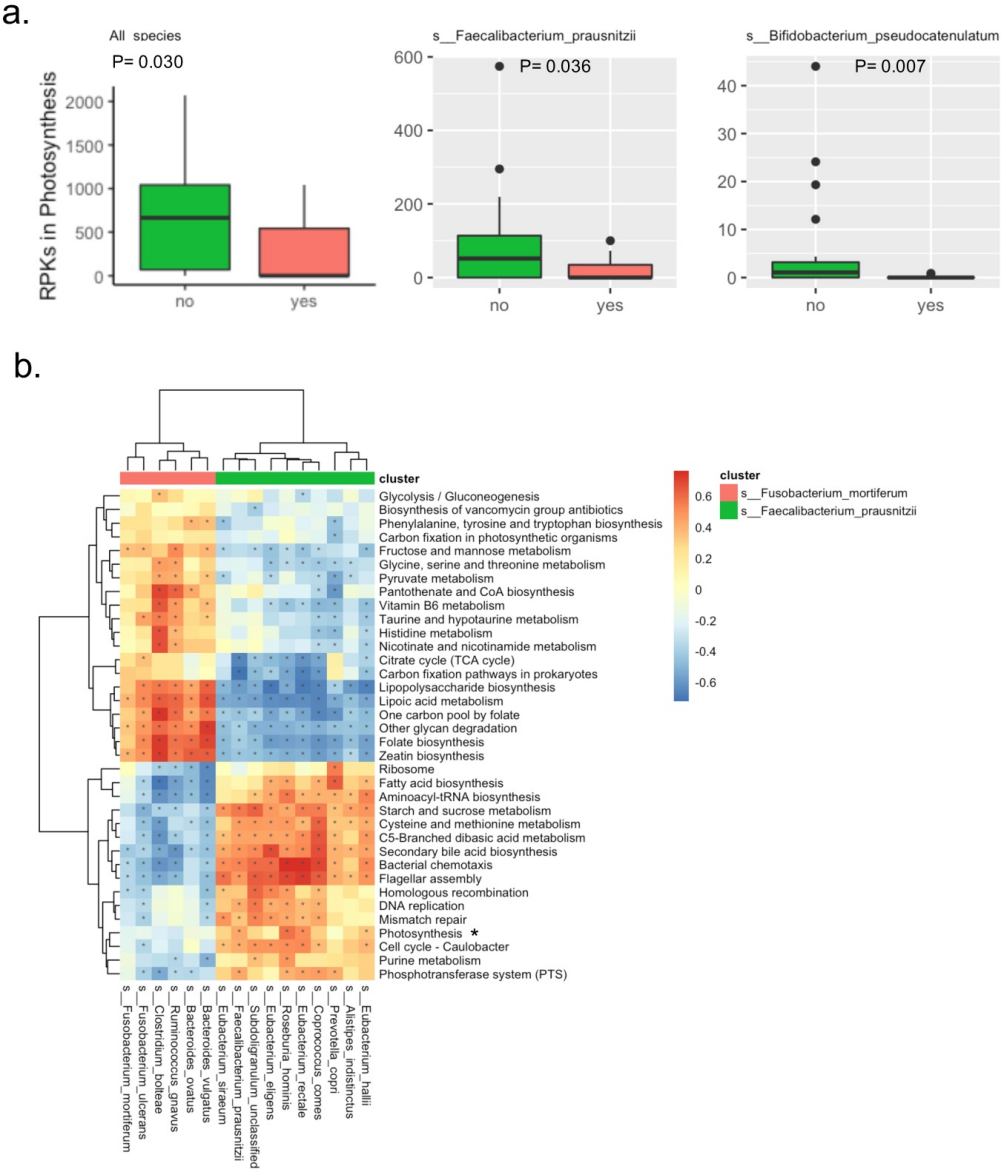
** Bacterial KEGG pathways in the fecal samples from patients with intestinal adenomatous polyps.** (a). The RPKs in the Photosynthesis pathway were significantly different between the APC mutation group (red) and the non-APC mutation group (green). The RPKs in the Photosynthesis were mainly derived from two species: s__Faecalibacterium_prausnitzii and s__Bifidobacterium_pseudocatenulatum. The P values were obtained by Wilcoxon rank-sum test. RPKs: reads per kilobase. (b). Spearman correlation of relative abundance between the KEGG pathways and species. There were two clusters of species, in which the red cluster included s__Fusobacterium_mortiferum, the green cluster included s__Faecalibacterium_prausnitzii. The Photosynthesis pathway was highlighted because its relative abundance was significantly different between the APC mutation group and the non-APC mutation group. In the heatmap, color presented the correlation coefficient, and star indicated the correlation coefficient >0.25 and P value <0.05.

There were a total of 138 KEGG pathways identified by the detected microbial genes. The raw abundance of three pathways was significantly different between the two groups, in which the Bisphenol degradationpathway was only detected in the patients with APC mutation, the D-Arginine and D-ornithine metabolismpathway was significantly abundant in patients with APC mutation, while the Photosynthesis metabolismpathway was significantly abundant in patients without APC mutation. Interestingly in the Photosynthesis pathway, the microbial genes were mainly derived from two potentially beneficial species Faecalibacterium_prausnitzii and Bifidobacterium_pseudocatenulatum. The significantly lower abundance of the Photosynthesis pathway in patients with APC mutation was due to the lower abundance of species Faecalibacterium_prausnitzii and Bifidobacterium_pseudocatenulatum, suggesting the Photosynthesis pathway was a potentially beneficial pathway (Figure 2a). After filtering out very-low-abundance pathways, another KEGG pathway Phenylalanine metabolismwas detected to be significantly abundant in patients with APC mutation (Table S4). Random forest with the raw abundance of all the KEGG pathways had only 51% prediction accuracy for predicting APC mutation status. The top four most predictive KEGG pathways were D-Arginine and D-ornithine metabolism, DNA replication, Bacterial chemotaxis, Bisphenol degradation (Figure S6).

In the correlation analysis, two clusters of KEGG pathways were potentially correlated with two clusters of species characterized by Faecalibacterium_prausnitziiand Fusobacterium_mortiferum(Figure 2b). The beneficial speciesFaecalibacterium_prausnitziiwas clustered with other species including Prevotella_copri, Eubacterium_siraeum, Alistipes_indistinctus, Eubacterium_hallii, Eubacterium_rectale, Coprococcus_comes, Roseburia_hominis, Eubacterium_eligens and Subdoligranulum_unclassified; this potentially beneficial cluster of species were positively correlated with 16 KEGG pathways that were potentially beneficial as well, while negatively correlated with 20 KEGG pathways that were probably harmful. Specifically, the Faecalibacterium_prausnitziiwas positively correlated with Photosynthesis pathway discovered abovewith statistical significance. Similarly, theharmful species Fusobacterium_mortiferumwas clustered with other species including Ruminococcus_gnavus, Bacteroides_ovatus, Bacteroides_vulgatus and Clostridium_bolteae; this potentially harmful cluster of species was positively correlated with the probably harmful cluster of 20 KEGG pathways mentioned above, while negatively correlated with the potentially beneficial cluster of 16 KEGG mentioned above.

There were a total of 138 MetaCyc pathways identified by the detected microbial genes. The prediction accuracy for predicting the APC mutation status by MetaCyc pathways was only 50%. The most important one was acetyl-CoA fermentation to butanoate II, followed by pyruvate fermentation to acetone (Figure S7a). A total of 5 MetaCyc pathways were significantly different in patients with APC mutation and patients without APC mutation group. The pathways including 1,4-dihydroxy-6-naphthoate biosynthesis II, pyruvate fermentation to acetone, acetyl-CoA fermentation to butanoate II and UMP biosynthesis were significantly abundant in patients without APC mutation while theformaldehyde oxidation I pathway was significantly abundant in patients with APC mutation (Figure S7b). Similarly, the correlation analysis of the relative abundance of MetaCyc pathways and species detected the same two clusters of species characterized by Faecalibacterium_prausnitziiand Fusobacterium_mortiferum(Figure S8).

### Analysis of Serum Metabolites and APC mutation

**Figure 3:**
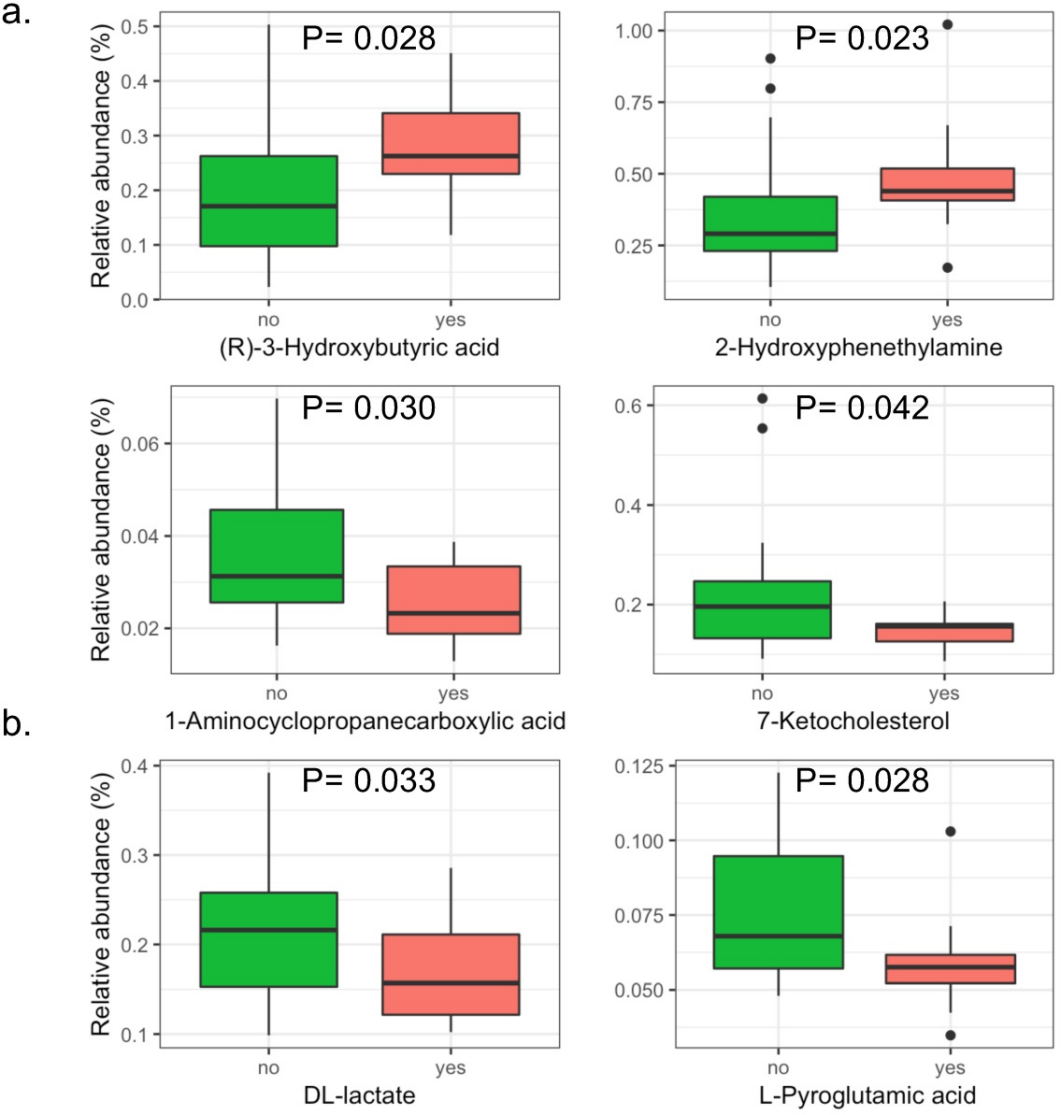
** The significant serum metabolites in patients with intestinal adenomatous polyps.** The case group is the APC gene mutation group (red) and the control group is non-APC gene mutation group (green). (a). The positive iron model. (b). The negative iron model. The y axis shows the relative abundance of each serum metabolites. The P values were obtained by Wilcoxon rank-sum test.

A total of 146 metabolites in the positive iron model and 151 metabolites in the negative iron model were identified in the serum of patients with intestinal adenomatous polyps. The PLS-DA score plots showed that in the baseline, the serum metabolites in the two groups was not significantly separated either in the positive or negativeiron model (Figure S9). Overall, there were four metabolites significantly abundant in patients without APC mutation, including 1-Aminocyclopropanecarboxylic acid, 7-Ketocholesterol, DL-lactate andL-Pyroglutamic acid, and two metabolites significantly abundant in patients with APC mutation, including (R)-3-Hydroxybutyric acid and 2-Hydroxyphenethylamine (Figure 3).

The risk index for each patient was generated from the relative abundance of the six significant serum metabolites, or the four significant species discovered above. The patients with APC mutation could be distinguished from the patients without APC mutation, using the risk index generated from species (AUC=83.22%) or metabolites (AUC=86.71%), although the differencewas not statistically significant (Figure S10, P=0.48).

**Figure 4:**
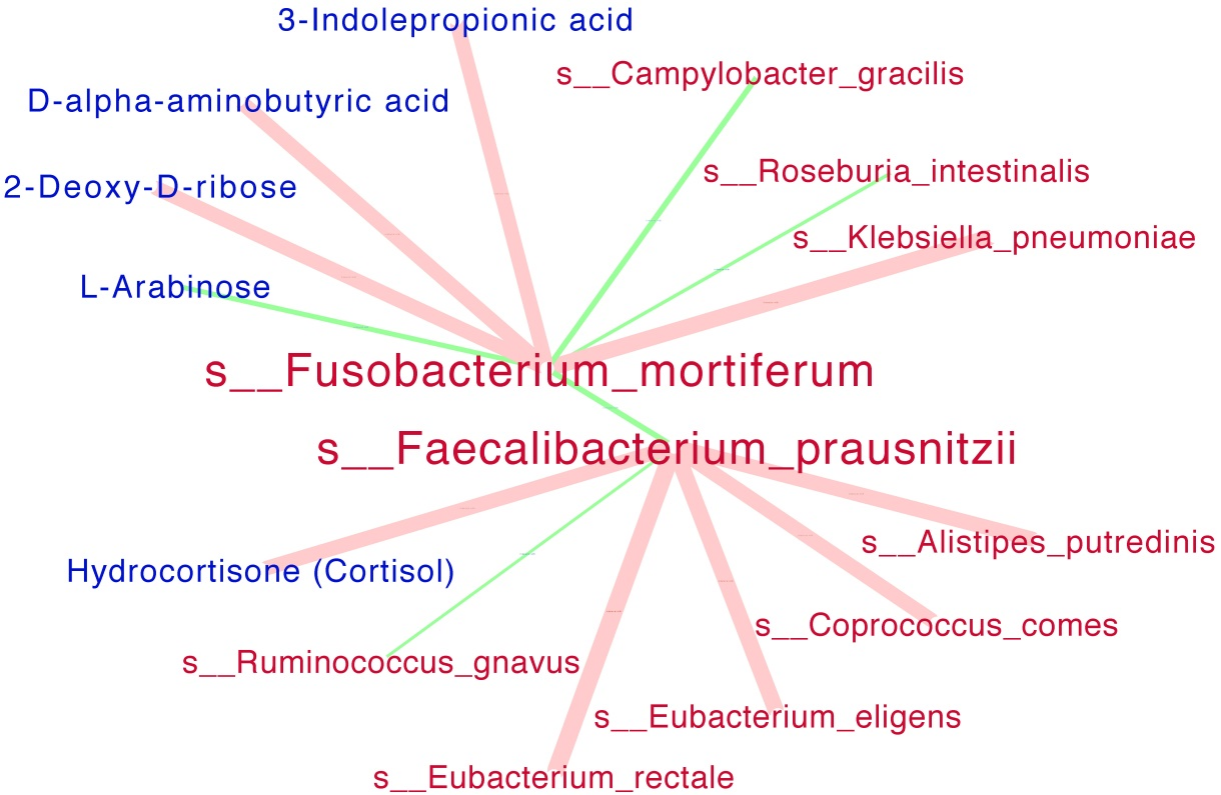
** Interaction networks among significant species and metabolites.** SparCC analysis was run simultaneously for all the species (red) and serum metabolites (blue). The size of each edge presented the Spearman correlation coefficients. Positive correlations were indicated as red edges and inverse correlations as green edges (SparCC R > = 0.25, P < = 0.05 for displayed edges). Note: only the networks for s__Fusobacterium_mortiferum and s__Faecalibacterium_prausnitzii were showed.

Interaction networks among significant species and serum metabolites were presented in Figure 4. The beneficial specie Faecalibacterium_prausnitzii was negatively correlated the harmful species Fusobacterium_mortiferum with statistical significance. In details, the Faecalibacterium_prausnitziiwas positively correlated with Hydrocortisone (Cortisol) metabolite while negatively correlated with Ruminococcus_gnavus; the Fusobacterium_mortiferum was positively correlated with Klebsiella_pneumoniae while negatively correlated with L-Arabinose metabolite.

## Discussion

Given the increasing role of gut microbiome in CRC pathogenesis, we aimed to investigatethe association of highly pathogenic APC mutation with gut microbiomes and explore the characteristics of serum metabolites in patients. Firstly, we found that Fusobacterium_mortiferum was significantly increased in patients with APC mutations while Faecalibacterium_prausnitzii and Bifidobacterium_pseudocatenulatum were less abundant in patients with APC mutations. Moreover, the pathway analysis discovered two clusters of pathways and two clusters of microbiomes characterized by Fusobacterium_mortiferum and Faecalibacterium_prausnitzii, both of which were inversely correlated. Finally, some of the serum metabolites in patients with APC mutations were significantly different from patients without APC mutation; however, more evidence is needed to clarify the association between serum metabolites and gut microbiome.

On one hand, Faecalibacterium Prausnitzii, a well-known probiotic was enriched in patients without APC mutations in our study. It is the most important butyrate-producing bacteria in the human gut microbe and has been considered as a biological indicator of human health 17. In the intestine, Faecalibacterium prausnitzii produce energy and anti-inflammatory metabolites to colonocytes to maintain intestinal health. The most important ability of Faecalibacterium prausnitzii is to ferment glucose into acetate, butyrate, D-lactate and formate, in which the butyrate is generally considered to be a key metabolite for reducing the risk of CRC 18-20, probably suppressing chronic intestinal inflammation by regulating T cells 21. Moreover, Faecalibacte praiumnitzii can also produce another anti-inflammatory metabolite, salicylic acid, which can significantly reduce IL-8 levels *in vitro*^22^.In conclusion, Faecalibacterium prausnitzii is an important anti-inflammatory microbiome known in the intestine, and more and more studies have linked it to CRC 23.In our study, the serum metabolite hydrocortisone (Cortisol) showed a significant positive correlation with Faecalibacterium Prausnitzii. Cortisol is a steroid hormone of the glucocorticoid class and functions to increase blood sugar through gluconeogenesis, to suppress the immune system, and to aid in the metabolism of fat, protein, and carbohydrates. To our best knowledge, the relationship between the serum concentration of Cortisol and the abundance of Faecalibacterium Prausnitzii in the human gut has not been previously shown. Bifidobacterium pseudocatenulatum was another microbiome enriched in patients without APC mutations in our study. The Bifidobacterium has the potential to restore the balance of lymphocytes and macrophages, so its absence may break the balance of the original intestinal immune system, triggering inflammation and destroying intestinal epithelial cells 24.

On the other hand, Fusobacterium mortiferum, which was enriched in patients with APC mutations in our study, has been previously shown to be correlated with higher occurrence of CRC 25-29. For example, a recent experiment on a mouse model of CRC revealed that Fusobacterium may be involved in distant metastasis of CRC 30. In addition, many other studies have found the elevated Fusobacterium abundance and lower T cell infiltration[31]and poor survival 32.Compared to the species Fusobacterium nucleatum as a potential biomarker to predict CRC^29^, ^33^, another species Fusobacterium mortiferumhas not attracted widespread attention. In 2005, a case report described a rare case of thyroid abscess caused by mixed anaerobic flora containing Fusobacterium mortiferum. In our study, Fusobacterium mortiferum was significantly correlated with four metabolites. Of notice, 2-Deoxy-D-Ribose, which was significantly positively correlated with Fusobacterium mortiferum, has been reported to participate in the development of human tumors by promoting angiogenesis 34. Therefore, we can deduce that the highly pathogenic APC mutation may be characterized by some specific microbiome, or associated with the abnormal inflammatory response of the intestine.

In our pathway analysis, the abundance of Photosynthesis pathway was significantly reduced in the APC-mutated patients, suggesting that the energy preservation in the way of ATP generation was lower, while energy utilization might be higher in these patients. This finding indirectly supports the development of CRC from APC-mutated intestinal adenomatous polyps is a high energy-consuming process. Moreover, the microbial genes in the Photosynthesis pathway were primary from two beneficial microbiomesFaecalibacterium_prausnitzii and Bifidobacterium_pseudocatenulatum. In our correlation analysis, we discovered the Faecalibacterium_prausnitzii was positively correlated with Photosynthesis pathway significantly, and it was clustered with other microbiomes sharing the similar pattern of correlation with other pathways.

The pathway analysis also indicated the abundance of both D-Arginine and D-ornithine metabolism pathway and Bisphenol degradation pathway were significantly higher in patients with APC mutations. Few evidence was available tosupport the association between either D-Arginine, D-ornithine and intestinal diseases. But it has been reported that the exposure to dietary bisphenol A (BPA) 35 or its interaction with other ingredients 36 may have aneugenic effects on colon cancer cells by disrupting the normal gut microbiota. Furthermore, the xenoestrogen BPA at nanomolar and greater concentrations could even modulate the protein profiles and promote the metastasis of CRC cells via induction of mesenchymal transitions 37. Our data showed that the pathway of Bisphenol degradation might be significantly enhanced in the APC mutation group, implicating higher concentrations of BPA in these APC-mutated patients with intestinal adenomatous polyps. Therefore, the microbiomes involved may include the beneficial microbiomeBifidobacterium, as it has been reported that the proportion of the Bifidobacterium was elevated in fecal samples of BPA-exposed females 33.

It has been well-known that short-chain fatty acids (SCFAs) play a key role in the prevention and treatment of CRC^38^-^40^, which is indirectly supported by our findings. As our pathway analysis showed, the fatty acid biosynthesis pathway was positively correlated with the microbiome cluster characterized by Faecalibacterium_prausnitzii, while negatively correlated with the microbiome cluster characterized by Fusobacterium_nucleatum. Moreover, another SCFAs-related pathway fermentation of pyruvate wassignificantly abundant in the APC-mutated patients with intestinal adenomatous polyps, implying a high level of pyruvate. Because CRC cells could acquire the ability to escape from the cell death either by APC gene mutation 41 or by maintaining low levels of pyruvate 42, our finding suggests that the APC-mutated adenoma cells may interact with SCFAs to avoid cell death.

Cumulative evidence suggests a link between the gut microbiome and the body's metabolic health 43-46. A latest study applying metagenomic and metabolomic analyses to CRC with different stages has indicated that the gut microbiome presented stage-specific phenotypes 47. Although extensive studies of gut microbiome for either colorectal adenoma or CRC have consistently identified some specific species as Fusobacterium_nucleatum, few evidences was available to characterize the gut microbiome in the specific patients, especially the patients with APC-mutated colorectal adenoma, or study its association with serum metabolites. Our study discovered one beneficial cluster of species by Faecalibacterium_prausnitzii, and its abundance was positively correlated with a steroid hormone Hydrocortisone (Cortisol). As glucocorticoids could promote the arginine metabolism in enterocytes 48, our discovery of a lower abundance of Faecalibacterium_prausnitzii and a lower level of serum Hydrocortisone (Cortisol) may imply inactivated metabolism of arginine. However, we discovered a higher abundance of arginine metabolism pathway in these patients. Since arginine metabolism was well accepted to play an important role in cancer cells development, we hypothesis that enhanced metabolism of arginine in patients with APC-mutated colorectal adenoma was probably due to other microbiomes.

It has been reported that the alteration of microbiota is closely related to CRC based upon their toxic and genotoxic metabolites produced by fermentation of dietary ingredients 49.In our network analysis of species and serum metabolites, three metabolites were positively correlated with Fusobacterium_mortiferum, in which the 3-Indolepropionic acid has the potential to protect primary neurons and neuroblastoma cells against oxidative damage and death, but its role in the colon has not been studied^50^. Another significant metabolite was D-alpha-aminobutyric acid, which was the substrate of D-amino acid oxidase 51. The metabolic formation of alpha-aminobutyric acid from methionine may involve the paradigm of the 'adenoma-carcinoma sequence', since the methionine can regulate the innate immune system and promote the tumor-Initiating cells 52-54. Another metabolite positively correlated with Fusobacterium_mortiferum was 2-deoxy-D-ribose. Although the 2-deoxy-D-ribose has the potential to promote angiogenesis 55, but its relationship with gut microbiome or its role in colorectal adenoma or cancer has not been studied either. In our study, the serum level of L-Arabinose was negatively correlated with Fusobacterium_mortiferum abundance. The arabinose was potentially related to gut microbiome as the accumulated arabinose from the cecum to the colon was only detected in specific pathogen-free mice but not germ-free mice 56. Although L-Arabinose has the potential to lower inflammation in CRC cells, but its relationship with gut microbiome was not clearly studied 57.

## Conclusion

In our study, three species Bifidobacterium Pseudocatenulatum, Faecalibacterium Prausnitzii and Fusobacterium mortiferum were potentially related to the APC gene mutation status in patients with intestinal adenomatous polyps. Although our study showed the significant association between abundance of Faecalibacterium_prausnitzii and serum level of Hydrocortisone (Cortisol) by using SparCC analysis, the further in-depth experiments are needed to explore and demonstrate the biological function of gut microbiome in patients with APC-mutated intestinal adenomatous polyps.

## Supplementary Material

Supplementary Figure S.

Table S1.

Table S2.

Table S3.

Table S4.

## Figures and Tables

**Table 1:** characteristics of the patients with intestinal adenomatous polyps

	control	case	P
N	22	13	
gender			
Male	11 (50%)	9 (69%)	0.449
Female	11 (50%)	4 (31%)	
age (years)	59.05±12.05	60.15±14.85	0.959
body mass index (kg/m^2^)	23.74±4.77	24.12±6.34	0.528

The case group is the APC gene mutation group and the control group is non-APC gene mutation group.
