# Supplementary material for: Gut microbiome associated with APC gene mutation in patients with intestinal adenomatous polyps
Source: Int J Biol Sci. 2020 Jan 1;16(1):135–46. doi: 10.7150/ijbs.37399 (PMC6930378; doi:10.7150/ijbs.37399)
Supplement: Supplementary file 1 — Supplementary Figure S. [file ijbsv16p0135s1.pdf]

Supplementary table legends:

Table S1: The clinicaldetails for each patient

Table S2: The 76 exon amplified regions of APC gene

Table S3: The amount of sequence collected for each patient

Supplementary Figures:

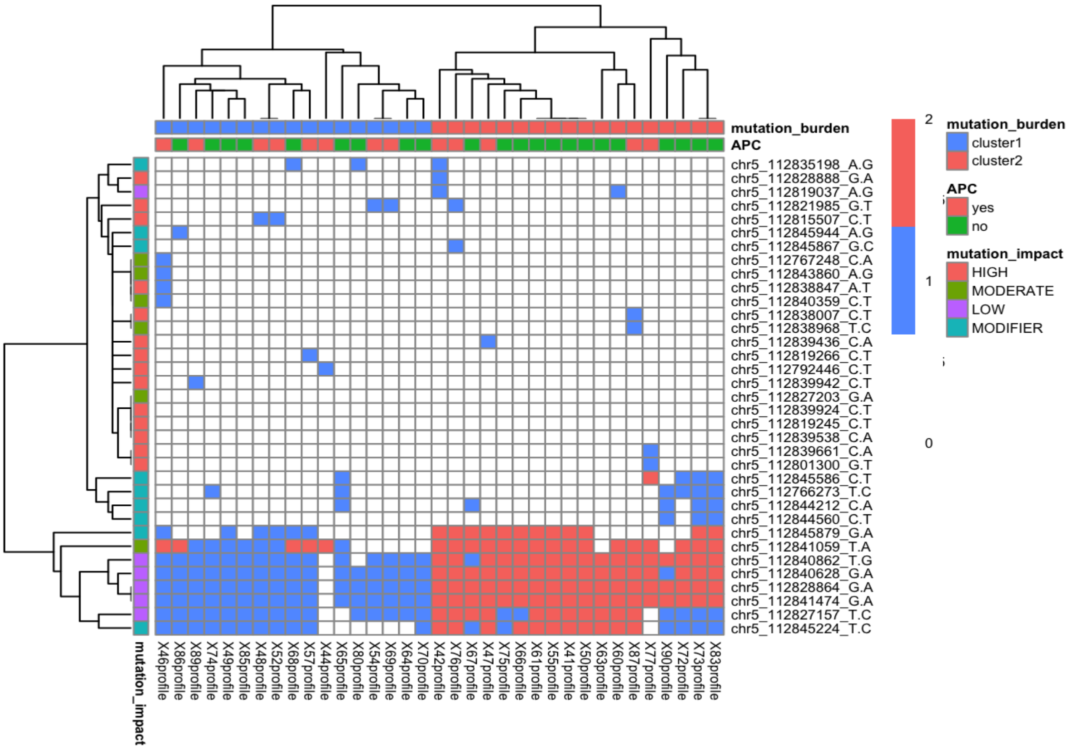

Figure S1. The raw mutation of sequencing exons in the APC gene among the patients with intestinal adenomatous polyps. The potential function of the mutated variants were identified by Variant Effect Predictor, and divided into four types (high, moderate, low and modifier). Only the highly pathogenic mutation was assumed to have high impact in the protein function, therefore, the patients were divided into two groups based on the presence (red) or absence (green) of highly pathogenic mutations. In the heatmap, the rows present the patients, while the columns present the APC exons. For example, the chr5_112835198_A.G stands for base pair at 112835198 on chromosome 5 in the the reference genome GRCh38, the reference allele is A, and the alternative allele is G. The colors present the number of

alternative alleles, thus indicate the wild-type (white), heterozygous mutated (blue) and homozygous mutated (red).

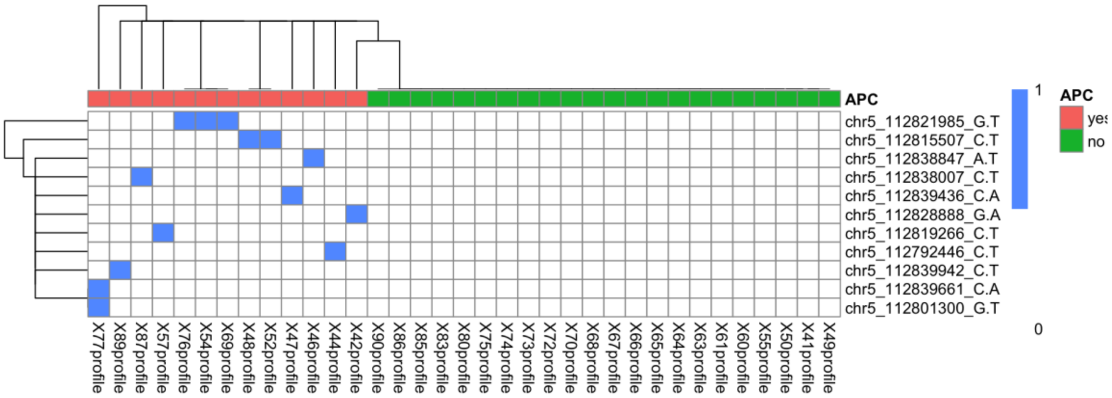

Figure S2. The patients with intestinal adenomatous polyps were divided into two groups by the status of highly pathogenic APC gene mutation. The case group is the APC gene mutation group (red) and the control group is non-APC gene mutation group (green). In the heatmap, the rows present the patients, while the columns present the APC exons. The colors stand for the presence (blue) or absence (white) of the highly pathogenic exon.

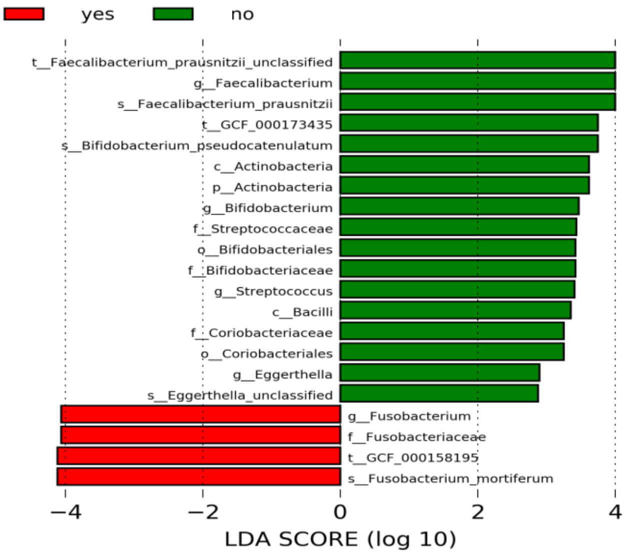

Figure S3. LEfSe analysis of fecal microbiomes in patients with intestinal adenomatous polyps. The bacterial clades were differentially abundant between the APC gene mutation group (red) and non-APC gene mutation group (green). Clades in this graph were both statistically significant (P <0.05) and had an LDA score >±4, considered a significant effect size. Prefix k__ is kingdom, p__ phyla, c__ class, o__ order, f__ family, g__ genus, s__species and t__strain.

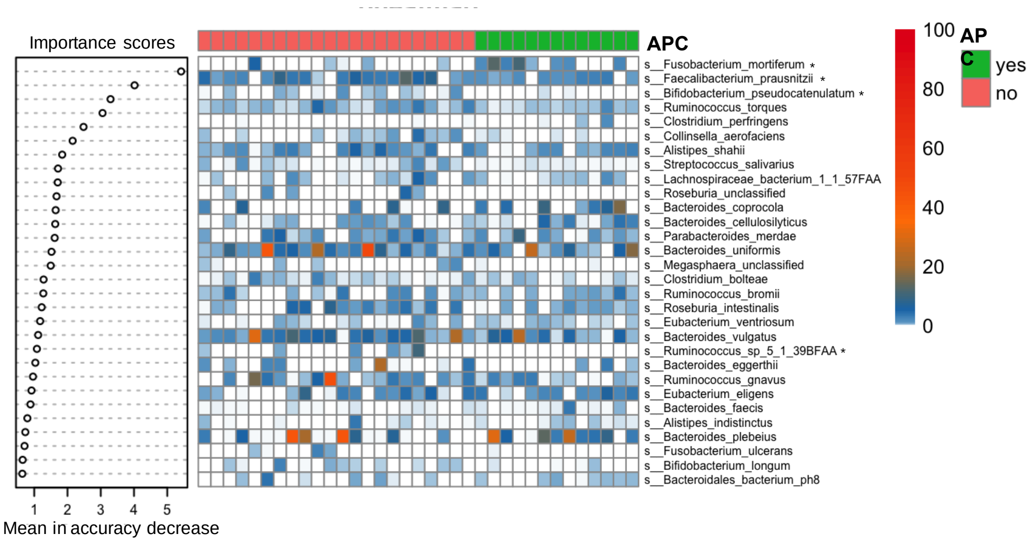

Figure S4. The importance and relative abundance of bacterial species in fecal samples of patients with intestinal adenomatous polyps, compared the APC mutation group to the no-APC mutation group. (left panel) Analysis using the machine learning algorithm Random Forest. Bacterial species that most strongly distinguish patients with APC mutation from those without APC mutation were identified. Importance scores were derived from the loss in accuracy measured when each indicated specie was removed from the analysis. The units on the x axis indicate mean decrease in accuracy. (right panel) A heatmap demonstrating relative abundance of bacterial species in patients with APC mutation and no-APC mutation. The * indicated the species that were statistically different in abundance between two

groups by Wilcoxon rank-sum test.

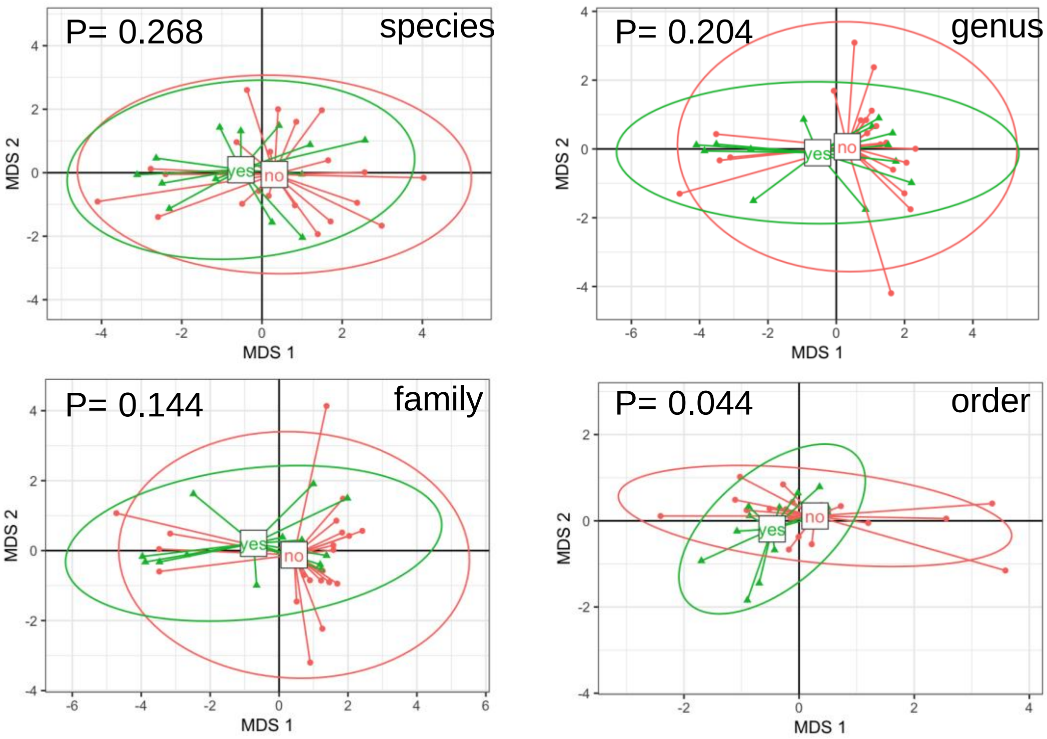

Figure S5. Cluster analysis based on the multidimensional scaling (MDS) of the Jensen-Shannon distance using the relative abundance of all the fecal microbiomes in the taxanomic level of species, genus, family or order. Colors indicated the APC mutation group (green) or non-APC mutation group (red). The P values were obtained by the permutational multivariate analysis of variance.

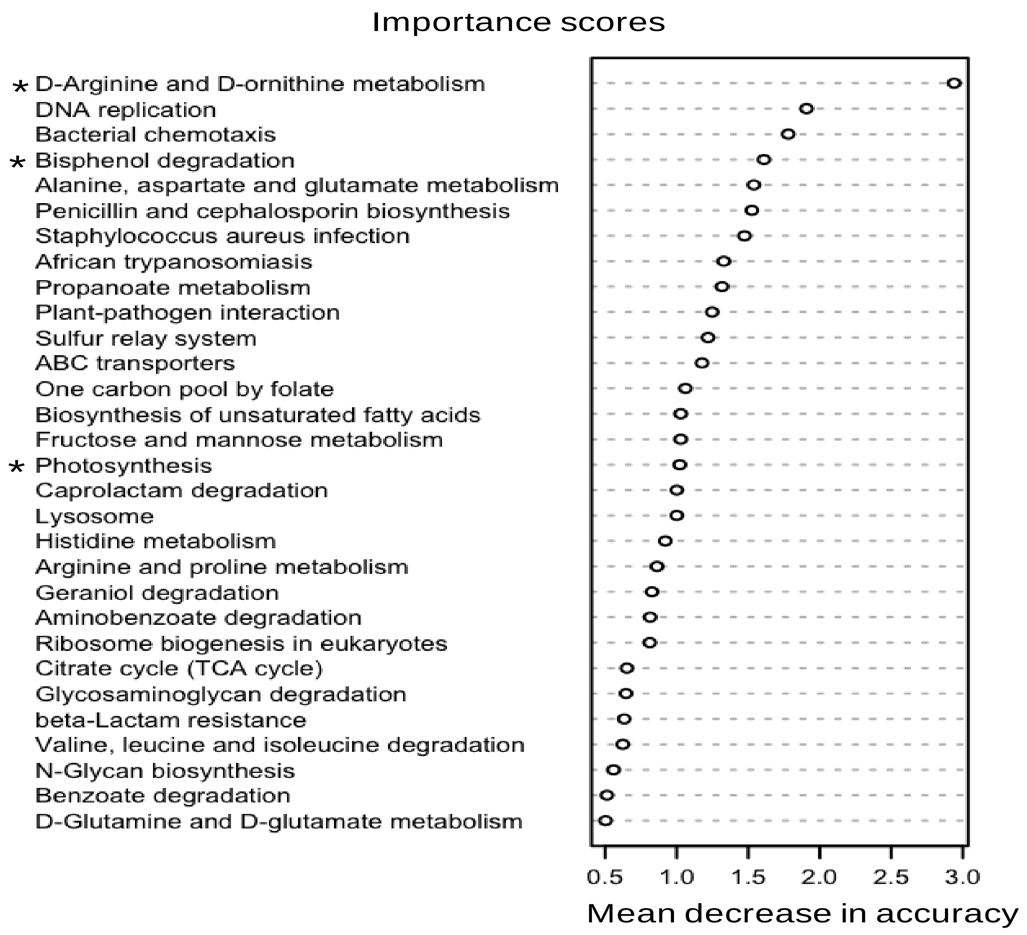

Figure S6. The machine learning algorithm Random Forest was used to identify the KEGG pathways that most strongly distinguish the two groups. The raw abundance of three KEGG pathways were significantly different between the two groups, indicated by the stars.

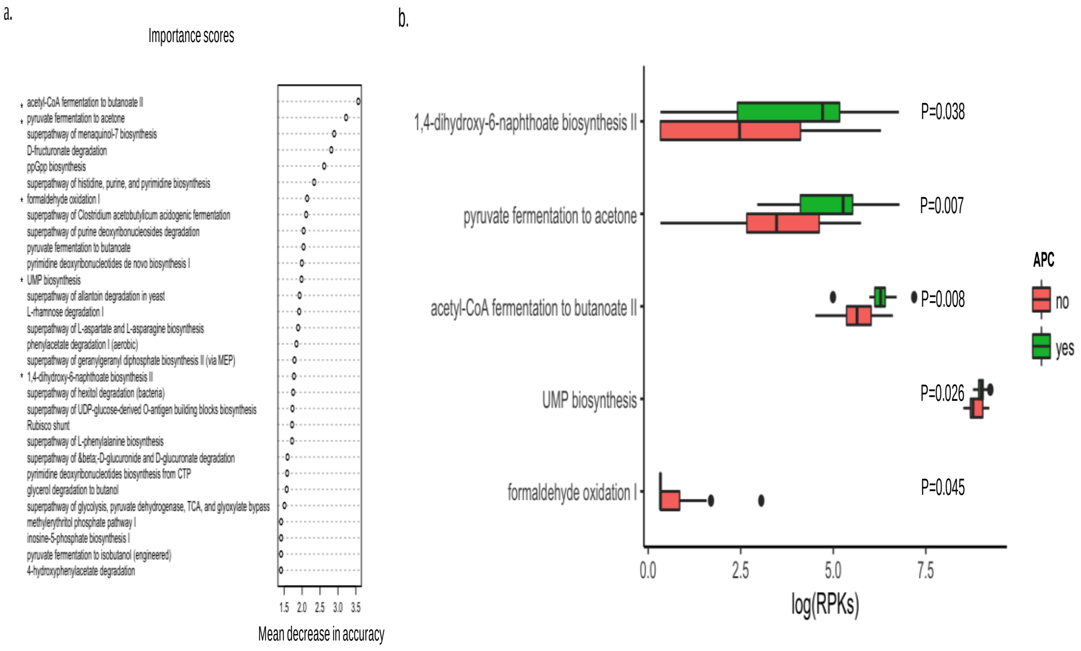

Figure S7. Association between MetaCyc pathways and APC mutation status in patients with intestinal adenomatous polyps. (a.) The machine learning algorithm Random Forest was used to identify the MetaCyc pathway that most strongly distinguish the patients with intestinal adenomatous polypswith APC mutation or not. The raw abundance of 5 MetCyc pathways were significantly different between the two groups, indicated by the stars. (b.) The significant MetaCyc pathways identified in fecal microbiomes in patients with intestinal adenomatous polyps. The status of APC mutation was indicated by two colors. The x axis presented the log transformation of reads per kb (RPKs) of target DNA in the samples. The P values were obtained by Wilcoxon rank-sum test.

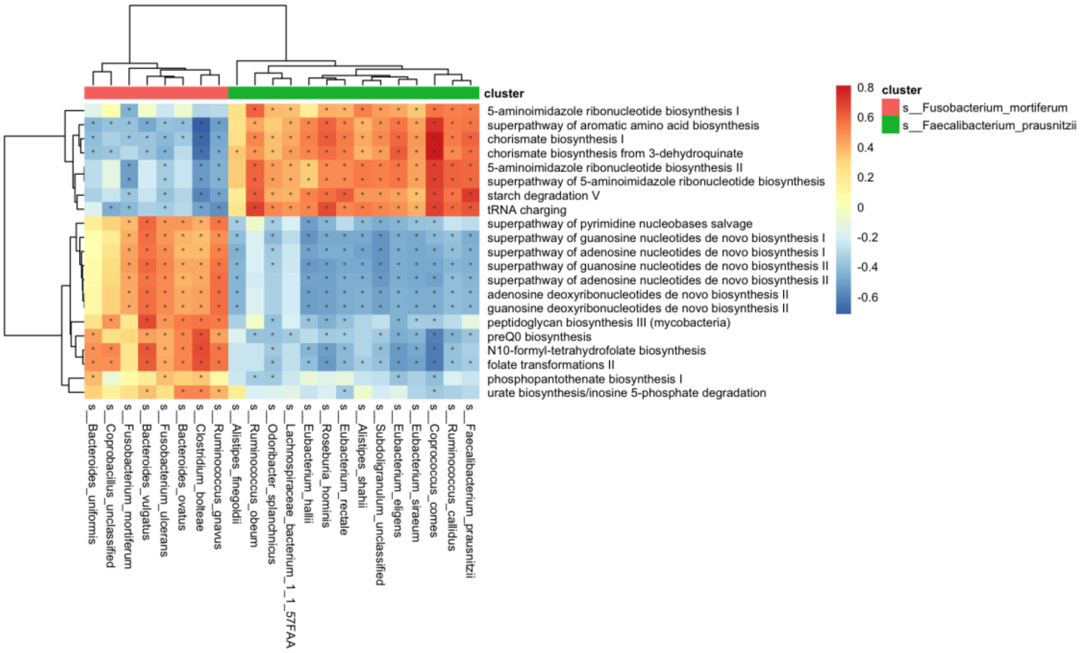

FigureS8. Bacterial MYC pathways in the fecal samples from patients with intestinal adenomatous polyps. Spearman correlation of relative abundance between the MYC pathways and species. There were two clusters of species, in which the red cluster included s__Fusobacterium_mortiferum, the green cluster included s__Faecalibacterium_prausnitzii. In the heatmap, color presented the correlation coefficient, and star indicated the correlation coefficient >0.25 and P value <0.05.

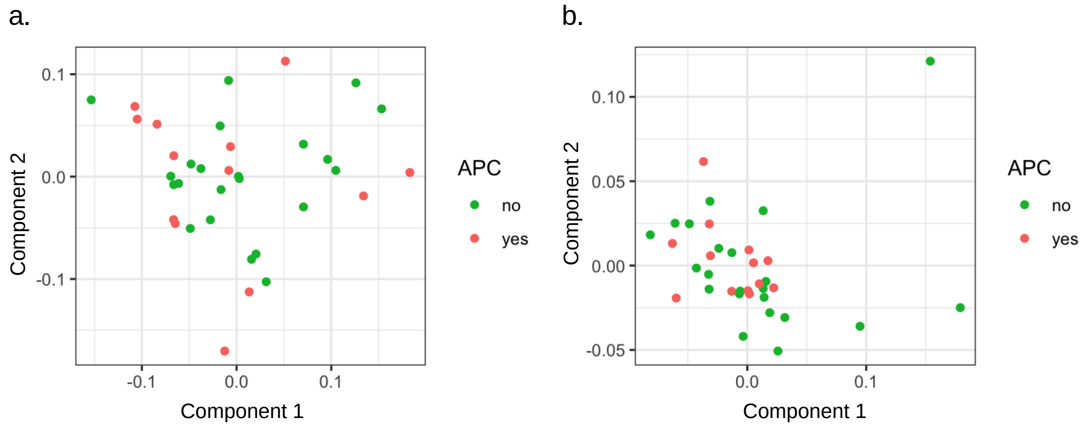

Figure S9. The PLS-DA score plots from the analysis of serum metabolites. (a). The positive iron model (P=0.78), (b). The negative iron model (P=0.48). The P values were obtained from the permutational multivariate analysis of variance.

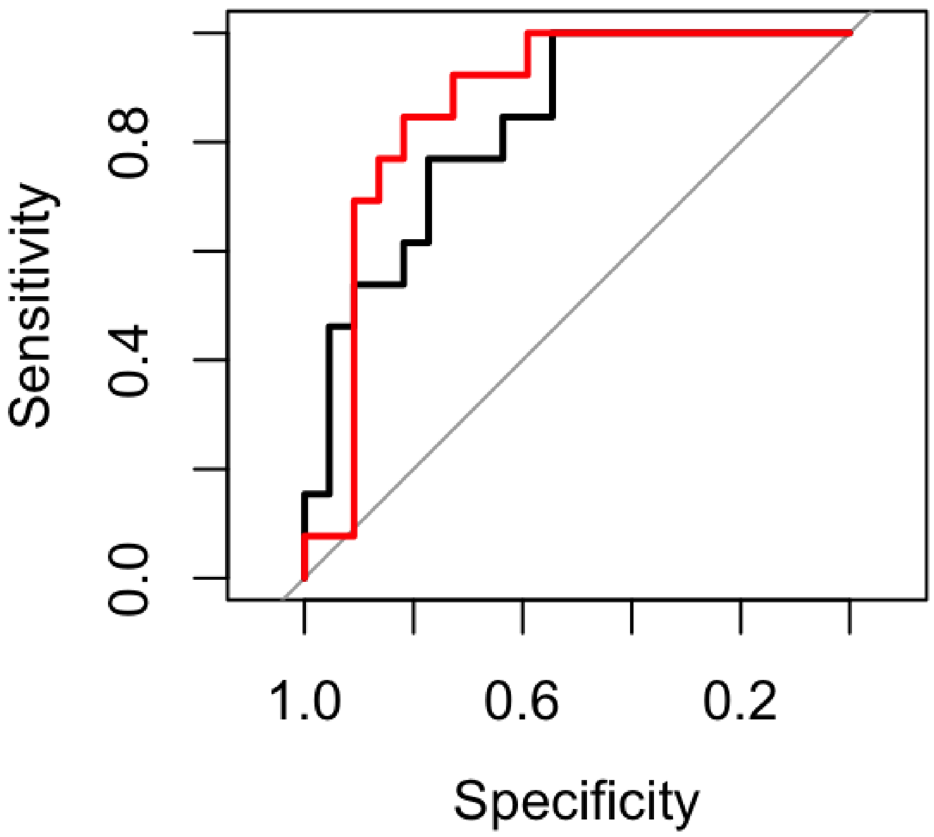

FigureS10. ROC curves in predicting the highly pathological mutation of APC gene by risk index obtained from significant fecal microbiomes and serum metabolites. The AUC for microbiome was 83.22% (black curve), while AUC for metabolites was 86.71% (red curve, P=0.48).The case group was the APC gene mutation group, while the control group was non-APC gene mutation group. The abundant microbiome or metabolites in control group were considered to be beneficial bugs, while abundant ones in case group were considered as harmful bugs. The difference between the sums of harmful bugs and the sums of beneficial bugs was calculated as the risk index value. ROC: receiver operating characteristic; AUC: area under the curve.
